# Reward cues readily direct monkeys’ auditory performance resulting in broad auditory cortex modulation and interaction with sites along cholinergic and dopaminergic pathways

**DOI:** 10.1038/s41598-019-38833-y

**Published:** 2019-02-28

**Authors:** Patrik Wikman, Teemu Rinne, Christopher I. Petkov

**Affiliations:** 1 Department of Psychology and Logopedics, University of Helsinki, 00014 Helsinki, Finland; 2 Turku Brain and Mind Center, Department of Clinical Medicine, University of Turku, 20014 Turku, Finland; 3 Institute of Neuroscience, Newcastle University, NE1 7RU Newcastle upon Tyne, United Kingdom; 4 Centre for Behaviour and Evolution, Newcastle University, NE1 7RU Newcastle upon Tyne, United Kingdom

## Abstract

In natural settings, the prospect of reward often influences the focus of our attention, but how cognitive and motivational systems influence sensory cortex is not well understood. Also, challenges in training nonhuman animals on cognitive tasks complicate cross-species comparisons and interpreting results on the neurobiological bases of cognition. Incentivized attention tasks could expedite training and evaluate the impact of attention on sensory cortex. Here we develop an Incentivized Attention Paradigm (IAP) and use it to show that macaque monkeys readily learn to use auditory or visual reward cues, drastically influencing their performance within a simple auditory task. Next, this paradigm was used with functional neuroimaging to measure activation modulation in the monkey auditory cortex. The results show modulation of extensive auditory cortical regions throughout primary and non-primary regions, which although a hallmark of attentional modulation in human auditory cortex, has not been studied or observed as broadly in prior data from nonhuman animals. Psycho-physiological interactions were identified between the observed auditory cortex effects and regions including basal forebrain sites along acetylcholinergic and dopaminergic pathways. The findings reveal the impact and regional interactions in the primate brain during an incentivized attention engaging auditory task.

## Introduction

Attention powerfully modulates brain activity in sensory cortices and selectively shapes neural responses in these regions^1–8^. However, due to a paucity of cross-species comparisons on attention-dependent modulations using comparable neurobiological measures, it is poorly understood how the neurocognitive systems in human and nonhuman animals compare. Recently, some authors have questioned the correspondence between monkey and human cognitive systems^9^, including the one involved in auditory cognition^10,11^. These concerns impede translating neuronal-level insights from nonhuman animal models to humans. While humans can be readily instructed to perform cognitive tasks, training nonhuman animals on such tasks, particularly in the auditory modality, is extremely challenging and time-consuming. Moreover, even after extensive training, lapses in attention occur in the nonhuman animals that alter neuronal responses^12^ or activation patterns^13^, complicating cross-species comparisons. It is thus imperative to further develop engaging tasks on which nonhuman animals can be quickly trained and which allow manipulating attention.

Incentivized attention tasks could be developed to expedite training and evaluate the impact on sensory cortex by different cognitive control systems, which have traditionally been studied separately. Monetary incentive delay (MID) tasks have been used to study reward processing in humans^14^ and other animals^15^. These tasks have also been modified to modulate visual attention in humans^16,17^ and visual categorization in monkeys^18^. In the monkey visual categorization study by Minamimoto and colleagues (2010), monkeys were required to perform a simple visual task to receive a juice reward. The task consisted of withholding a response while a wait signal was presented (red dot) and to respond to a go signal (green dot). Then two different types of reward cues were incorporated: high reward (HiRe; e.g., picture of a dog) or low reward (LoRe; cat). The reward cues effectively manipulated the monkeys’ performance; monkeys had fewer errors and faster responses in trials with HiRe than LoRe cues. This performance difference indicated that the monkeys were able to discriminate the picture categories. Importantly, the results showed that the monkeys recognized the visual categories within a single testing session. Moreover, the simple task required no response selection as the task was identical on each trial.

**Figure 1:**
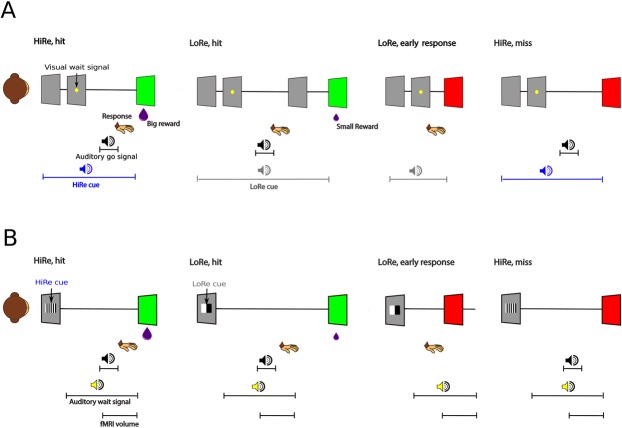
Auditory task with high or low reward auditory or visual cues: Incentivized Attention Paradigm (IAP). In all conditions, monkeys were required to withhold a response through a wait signal and to respond to an auditory go signal in order to receive a juice reward. In HiRe trials, a large reward (1 ml) was delivered immediately after a correct response. In LoRe trials, a small reward (0.1 ml) was delivered after a 7 s delay. In addition, visual feedback (green or red screen for correct or incorrect responses, respectively) was provided. (**A**) Four exemplary trials in auditory reward cue experiments (AudCue1 and AudCue2). If the monkey responded to the auditory go signal within a response window of 200–1300 ms in a HiRe trial, then a big juice reward was immediately delivered and the screen turned green. During LoRe trials, a correct response was associated with a delayed small reward and green screen. Note that the LoRe cue was presented until the reward was delivered. A response before the response window (early response) resulted in a red screen and trial termination. A red screen was also shown if the monkey did not respond before the end of the response window (miss). (**B**) Four exemplary trials in the fMRI experiment with visual reward cues and an auditory wait signal. See text and Table 1 for details.

Here we extended the paradigm used by Minamimoto and colleagues^18^ to modulate performance during a simple attention-engaging auditory task (Fig. 1) using auditory (AudCue1 and AudCue2 experiments) or visual cues (VisCue experiment). In the AudCue experiments, a visual wait signal and an auditory go signal were used. In the VisCue experiments, both wait and go signals were auditory. We hypothesized that (1) monkeys quickly discriminate between distinct auditory or visual HiRe and LoRe reward cues and use them to influence their performance during a simple auditory task, and that (2) fMRI would show activity modulation in broad regions of monkey auditory cortex, similar to auditory attention-related modulations reported in previous human imaging studies^1–3,13,19–21^. We found that both auditory and visual cues affected auditory task performance after minimal training (10 s to 100 s of trials; days or weeks, instead of months or years) with visual cues resulting in stronger effects. The fMRI results supported our initial hypotheses, revealing systematic modulations nearly exclusively in substantial portions of auditory cortex. Further analyses of psycho-physiological interactions (PPI) identified a number of regions that functionally interact with the auditory cortex effects, including basal forebrain sites involved in or influenced by acetylcholinergic and dopaminergic processes. The findings provide important insights into how an incentivized attention engaging auditory task influences the the sensory cortex in the primate brain.

## Materials and Methods

All of the nonhuman animal work and procedures described here were performed at Newcastle University and were approved by the Animal Welfare and Ethical Review Body at Newcastle University and by the UK Home Office. The work complies with the UK Animal Scientific Procedures Act (1986) and with the European Directive on the protection of animals used in research (2010/63/EU). We support the principles on reporting animal research stated in the consortium on Animal Research Reporting of *In Vivo* Experiments (ARRIVE). All persons involved in animal handling and procedures were certified and the work was strictly regulated by the UK Home Office.

### Macaque procedures

Three adult male rhesus monkeys (M1, M2, M3) from a group-housed colony were used for the auditory-cue experiments. At the beginning of the study, monkeys M1, M2 and M3 were 6, 6, and 8 years old and weighted 12.5, 11, and 10 kg, respectively. When all study components completed a year later, the monkeys were 12, 11, and 9.5 kg, respectively.

Two of the monkeys (M1, M2) also took part in the visual-cue experiment using fMRI. Given the ethical sensitivities involved in studying nonhuman primates and the 3Rs principles (one of which is on the Reduction of animal numbers), our work requires using the fewest macaques possible. A sample size of two to three is common in behavioral neuroscience experiments with macaques, provided that results are robust with each individual and that the effects generalize beyond one animal. Given that our results from several hundred trials with each animal are statistically robust and consistent in the overall pattern of effects between the animals there was no ethical justification to test additional monkeys.

Monkeys M1 and M2 had participated in a previous fMRI study and were already implanted with an MRI compatible head post for head immobilization during scanning (for details on the procedure, see^13,22^. Monkey M3 was previously trained to perform auditory tasks wearing a head-immobilizing face mask and helmet^23^. The quality of fMRI data that can be collected with the system is currently being assessed, but the system can already be used to collect auditory task performance data (see^23^), as was done with M3 on the behavioral auditory task here. All of the animals had been slowly acclimated with positive reinforcement training to work within a primate testing chair and to allow the required periods of head immobilization.

We relied on operant training with an individually customized fluid control procedure to ensure that the animals were motivated to work on the challenging tasks, while staying in a high state of wellbeing. This included using their preferred juice as reward to motivate them to perform the tasks (see^24^ for details). The animals had unrestricted access to fluid on days when they were not being trained or tested and over the weekends. Each daily (behavioral) training session was continued until the monkey’s motivation waned, and they stopped working.

### Behavioral experiments (AudCue1, AudCue2 and VisCue)

The monkeys were initially taught a simple auditory task. First, after 500 ms from trial onset a yellow filled circle (visual wait signal) appeared at the middle of the screen on grey background. The visual wait signal remained on the screen from 700 ms until the end of the trial (trial start and end are explained below). After a random interval of 500–1500 ms, an auditory go signal was presented. The auditory go signal was a 400 ms (including 8 ms onset and offset ramps) macaque “coo” vocalization recorded from a male macaque that was unfamiliar to the two individuals tested. If the monkey responded to the auditory go signal by pressing the lever within 200–1300 ms from its onset, then the response was accepted as a correct response and a juice reward was delivered. Incorrect responses (early responses during the visual wait signal) or missing the auditory go signal (no response before the end of the response window) were not rewarded, and the next trial started after a 200 ms delay. Visual feedback cues were used to supplement the juice reward during correct trials (green screen) or to emphasize an incorrect trial with no reward (red screen). A green screen was shown immediately after a correct response concurrently with reward delivery (200–1000 ms, depending on the size of the reward, see next paragraph). A red screen was shown for 200 ms immediately after an incorrect (early) response or at the end of the response window for miss trials. During this initial general task training in the laboratory, the monkeys mastered the simple auditory task in one session (ca. 500 trials).

Next, we acquired behavioral data while the monkeys performed the auditory task combined with auditory HiRe and LoRe cues. Since this was the first auditory study to use a version of the monetary incentive delay (MID) task, we did not know whether and which acoustical properties could be used to differentiate the cues. Therefore, we tested two sets of auditory cues and observed similar results with both. It is important to note that the monkeys were not explicitly trained to discriminate the HiRe and LoRe cues. In the AudCue1 experiment (Fig. 1A, Table 1), the HiRe cue consisted of narrow-band noise (bandpass filter centered at 2 kHz, 2 kHz bandwidth, 3 Hz sinusoidal amplitude modulation, 90% depth) and the LoRe cue was a simple tone (2 kHz sinusoid, 8 Hz amplitude modulation). In the AudCue2 experiment, we used a different set of auditory cues: the HiRe cue was a high-pitched sinusoidal tone (2 kHz, 8 Hz amplitude modulation) and the LoRe cue was a low-pitched tone (200 Hz, 3 Hz amplitude modulation). The HiRe cue was present in 50% of the trials and this cue indicated that a large reward (ca. 1 ml) would be delivered immediately after a correct response, whereas the LoRe cue indicated that a correct response would result in a small (ca. 0.1 ml) and delayed reward (7 s after the correct response). In all trials, either a HiRe or LoRe cue was presented from trial onset until the end of the trial. In HiRe trials, the trial terminated if the monkey gave a correct response (after immediate reward delivery) or if the monkey made an early response or if it did not respond before the end of the response window. LoRe trials were similar except that hit trials (correct response to the auditory go signal) continued until the delayed reward was delivered; this also helped to reinforce the association between the LoRe cue and the long delay. Note, however, that the response window was always 200–1300 ms from the onset of the go signal irrespective of the trial. The monkeys initiated each block of 20 trials by pressing and immediately releasing the lever. Within a block, the next trial started 200 ms after the completion of the prior trial. Monkeys M1 and M2 responded by pressing the response lever to start a block of 20 trials or to respond to stimuli. Monkey 3 used a different motor response contingency: pressing the lever to start each trial and releasing the lever to respond to stimuli.

**Table 1:** Events and timings (hit trials).

Condition	Event	Start time	Duration	Specification
AudCue1/AudCue2	HiRe cue	0 s	2.1–3.1 s	2 kHz (AM 3 Hz) band-pass noise/2 kHz (AM 8 Hz) tone
	LoRe cue	0 s	9.1–10.1 s	2 kHz (AM 8 Hz) tone/0.2 kHz (AM 3 Hz) tone
	Visual wait signal	0.5 s	0.7–2.6 s	Yellow fixation dot
	Auditory go nal	1–2 s	0.4 s	Macaque ‘coo’
VisCue	HiRe cue	0 s	2.1–3.1 s	High frequency pattern
	LoRe cue	0 s	9.1–10.1 s	Low frequency pattern
	Auditory wait signal	0.5 s	0.7–2.6 s	2 kHz (AM 8 Hz) tone
	Auditory go signal	1–2 s	0.4 s	4 kHz tone
fMRI_Low_/fMRI_High_	HiRe cue	0 s	5 s	High frequency pattern
	LoRe cue	0 s	12 s	Low frequency pattern
	Auditory wait signal	0.5 s	4.5 s	0.2 kHz (AM 3 Hz) tone/2 kHz (AM 8 Hz) tone
	Auditory go signal	2.3–3 s	0.4 s	4 kHz tone
	fMRI volume	3–5 s	2 s	

In the VisCue experiment, the auditory reward cues were replaced by visual reward cues, and the auditory go signal sound was a 4-kHz sinusoidal tone (duration 400 ms). Also, the visual wait signal (yellow filled circle) was replaced by an auditory wait signal (2-kHz tone, 8 Hz amplitude modulation). The VisCue behavioral study was conducted to see whether visual cues^18,25^ could be used to influence performance on an auditory task. The visual HiRe and LoRe cues consisted of high and low spatial frequency vertical gratings, respectively (Fig. 1B, Table 1).

In all behavioral experiments, the HiRe and LoRe trials were presented with equal probability in random order in runs of 100 trials. In one daily testing session, the monkeys completed 1–7 runs depending on their motivation. M1 and M2 testing started with the AudCue1 experiment, followed by the AudCue2 and VisCue experiments. M3 performed the AudCue experiments in reverse order. In AudCue1, M1 completed 30 runs (100 trials each), M2 25 runs, and M3 15 runs. In AudCue2, M1 completed 32 runs, M2 14 runs, and M3 15 runs. In VisCue, M1 and M2 completed 24 runs each.

Visual cues and visual wait signals were presented in the middle of a computer screen in front of the monkey (distance 1 m). All auditory signals were presented from two loudspeakers (Creative Inspire T10, distance 1 m, 30° to the left and right from the center of the screen; 65 dB SPL). The experiment was controlled using Cortex software (Salk Institute).

It is possible that in two of the three monkeys’ previous training history may have influenced their ability to pick up this task. Both monkeys (M1 and M2) had participated in our previous fMRI study^13^ on audio-visual attention in which they received extensive in daily sessions over two years on auditory or visual task training, while ignoring stimuli in the other modality. The final component of that study was attending to pictures and ignoring sound. However, M3 was previously trained only on an auditory task. This prior experience may have contributed to M1 and M2 showing stronger effects using visual cues, whereas M3 learnt to more readily use auditory cues (see Results).

### Analysis of Task Performance

Each trial was classified as a hit (correct response within the response window), early response (response before the response window), or miss (no response before the end of the response window). The mean hit rate (HR), early response rate (ER), miss rate (MR) and hit reaction times (RT) were calculated for each run separately for HiRe and LoRe trials. RT was calculated only for hits.

### fMRI experiment

The VisCue experiment was chosen for fMRI because it showed much stronger behavioral effects than the AudCue paradigms. This paradigm also had the advantage of having the same wait and go signals in HiRe and LoRe trials. For fMRI, the VisCue experiment was slightly modified to accommodate fMRI imaging timing constraints. Namely, in HiRe hit trials, reward delivery was delayed until after volume acquisition to avoid movement effects associated with juice consumption. Further, in hit trials, both the reward cue and auditory wait signals were always present until the end of the fMRI volume (5 s from the start of the trial) irrespective of whether the trial was a HiRe or LoRe hit trial. Also, if the monkey responded during the auditory wait signal but before the response window (early response) or missed the auditory go signal, a red screen was presented, and the trial terminated after the completion of the volume acquisition (auditory wait signal continued till the end of trial). These modifications were made so that irrespective of trial type (hit, miss or early response trial), the only stimulus level difference (before and during the fMRI volume acquisition) between Hire and LoRe trials was in the type of visual cue presented. Otherwise, the task, visual cues and auditory go signals were identical to those used in the behavior-only VisCue experiment (Fig. 1B, Table 1).

The auditory wait signal was either a low-pitched tone (0.2 kHz sinusoid, 3 Hz amplitude modulation; 50% of the runs) or a high-pitched tone (2 kHz sinusoid, 8 Hz amplitude modulation; 50% of the runs), and it was always played until the end of the MRI volume acquisition irrespective of the monkey’s behavior.

During fMRI, sounds were presented using MRI-compatible headphones (NordicNeuroLab) at 65 dB SPL (measured with an NTI Audio XL2 sound level meter). Visual stimuli were projected to a screen that the monkeys could see in a mirror in front of them. Scanner noise was attenuated with the ear cups around the headphones and acoustic noise dampening foam used around these (TempurPedic). The fMRI experiment was controlled using Cortex software (Salk Institute). The duration of the fMRI sessions was approximately 4 hours with preparation and take down, with the monkeys completing 2–5 runs of 100 trials per fMRI session.

HiRe and LoRe trials were presented with equal probability (40%) in a random order. During the remaining 20% of trials, no reward cues, nor auditory wait signals were presented but auditory go signals were present (this condition served as a baseline condition). The auditory wait signal was always the same within a session (either a high pitch sound or low pitch sound). Each monkey (M1, M2) completed 18 fMRI runs (each with 100 trials) with low (fMRI_low_) and 18 runs with high (fMRI_high_) auditory wait signals. For M1, we first completed data acquisition with fMRI_low_ followed by fMRI_high_. For M2 this testing order was reversed.

### MRI Procedures

The monkeys were scanned in a primate dedicated vertical 4.7 T MRI scanner (Brucker BioSpin, Etlingen, Germany). During data acquisition, the monkey sat in a scanner specific primate chair. Both monkeys (M1, M2) had been slowly acclimated to the scanner environment and having their head immobilized^13^. In each scanning trial, one fMRI volume was acquired 2500 ms after the onset of the auditory wait signals. That is, the volume was acquired during the rising edge of the expected peak of the BOLD response to the auditory wait signal^26^ (Fig. 1B, Table 1). In this way, the fMRI volume acquisition was positioned in time to largely capture the auditory response to the auditory wait signal, which is identical across all trials in each session.

Functional data were acquired using gradient-recalled echo planar imaging (EPI) sequence (TE 22 ms, volume acquisition time 2000 ms, flip angle 90°, matrix 96 × 96, FOV 9.6 × 9.6 cm^2^, slice thickness 2.0 mm with no gap, in-plane resolution 1 × 1 mm^2^, 20 axial slices covering most of the brain). Two structural scans were acquired in each session aligned with the functional volumes, which were used to help to register the functional volumes to the higher resolution anatomical image. One of these was a full-head EPI with extra slices. The other image was an anatomical volume (MDEFT; TE 6 ms; TR 20 ms; matrix 192 × 192, FOV 9.6 × 9.6 cm^2^, slice thickness 2.0 mm with no gap, in-plane resolution 0.5 × 0.5 mm^2^, 28 slices), which had higher in-plane resolution. Altogether 7200 functional volumes were acquired (2 monkeys × 2 to be attended sounds × 18 runs × 100 trials).

### fMRI data analysis

Global voxel-wise analysis was performed using FSL (version 5.8; www.fmrib.ox.ac.uk/fsl) separately for each run. The data were motion corrected, high-pass filtered (cutoff 100 s), and spatially smoothed (Gaussian kernel of 1 mm full-width half maximum). A general linear model with six explanatory variables (HiRe and LoRe trial; hit, early response or miss) was defined. The model also included four nuisance variables: trials after rewarded hit trials (to control for effects related to sensations and movements associated with the juice reward), reaction time for early response trials, reaction times for hit trials, and inter-volume interval (to model the effects of inter-image variation on the signal magnitude). In addition, 12 motion parameters were included in the model. Functional data of each scanning run were co-registered via the intermediate anatomical scans to a template monkey brain^27^ that is in register with a macaque brain atlas in stereotactic coordinates^28^.

Higher-level analysis was conducted across runs and animals. Using FreeSurfer tools (version 5.3, www.freesurfer.net), the contrast parameter estimates from the first level analysis were resampled to the cortical surface of the template monkey brain^27,29^ and smoothed on the surface (5 mm FWHM). Analysis across runs and monkeys was conducted using two-sided Welch’s *v* tests in surface space using FSL’s PALM^30^ (version alpha26; 10 000 permutations). The runs of one monkey were treated as a permutation and variance group to accommodate heteroscedasticity. Correction for multiple comparisons was performed using cluster mass correction (using PALM in FSL with a cluster defining threshold Z = 2.6, see^13,30^ for details) resulting in FWER corrected P values for each cluster (i.e., this procedure does not provide corrected P values for each node).

Regions of interest (ROIs) were defined by subdividing the superior temporal gyrus (STG) into 4 segments in the anterior-posterior direction, and ROI mean signal magnitudes were computed separately for each ROI and hemisphere.

### Group level behavioral and ROI analysis

**Figure 2:**
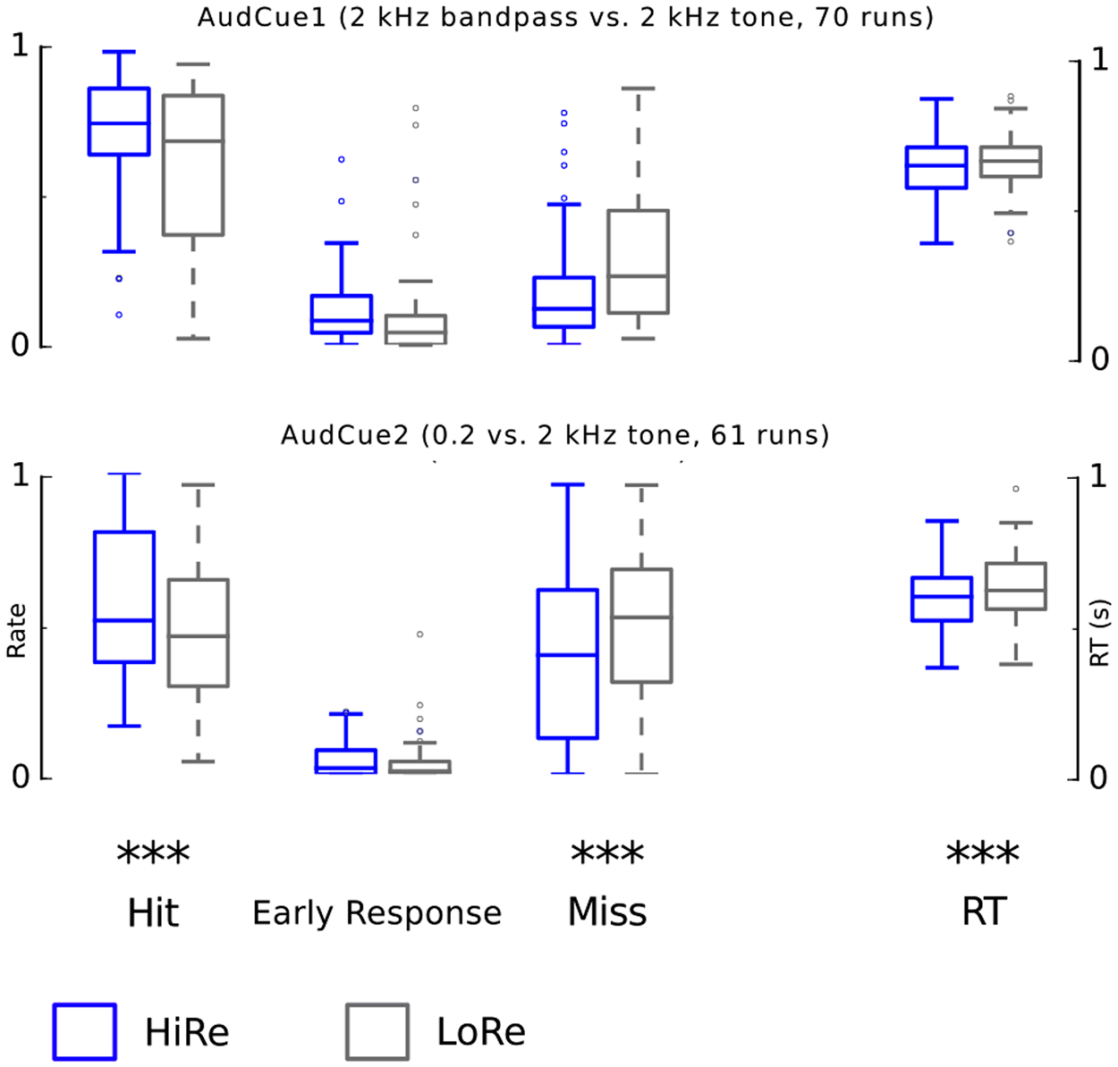
Performance in the AudCue behavioral experiments. Performance in AudCue1(top) and AudCue2 (bottom). The two leftmost box plots show mean hit rate across each run in the three monkeys for HiRe (blue) and LoRe (gray) trials. The other box plots show the early response rate, miss rate and reaction times (RT) correspondingly. The scale for RT is on the right side. Note that responses were classified as hit, early response or miss (i.e. in each run, HR + ER + MR = 1). Asterisks indicate significant differences between HiRe and LoRe trials [i.e. main effect of reward, *P < 0.05, **P < 0.01 and ***P < 0.001; AudCue: linear mixed model with factors reward (HiRe, LoRe), monkey (M1, M2, M3) and experiment (Audcue1, Audcue2)]. See results and Table 2 for details. The whiskers indicate 1.5 times the interquartile range (IQR) from the first and third quartile. The horizontal line inside the box indicates the sample median. Note that the significance values are for the repeated measures factor (reward cue), thus, for example, the variation in the difference score between HiRe and LoRe RT’s is smaller than one would expect from the box blots.

We used linear mixed models in SPSS to analyze the behavioral and ROI data (Figs 2, 4 and 6, Tables 2–4). These models included an intercept for run (each monkey had several runs). Reward (HiRe, LoRe) was treated as a repeated measures factor within each run. For each separate analysis, the covariance structure with the smallest Akaike Information Criterion was used. Permuted Welch’s *v* tests were used for pair-wise comparisons in Fig. 7. FWER correction was conducted across all pair-wise comparisons.

**Figure 3:**
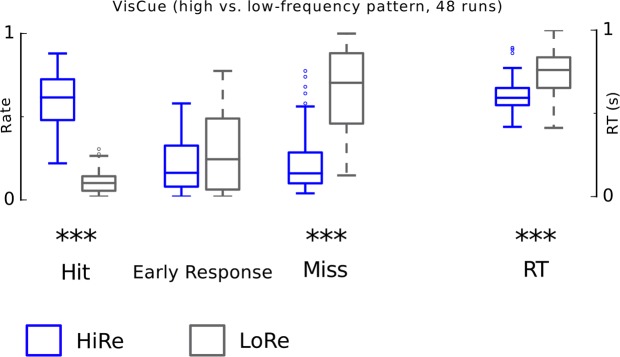
Performance in the VisCue behavioral experiment. Asterisks indicate significant differences between HiRe and LoRe trials [main effect of reward, *P < 0.05, **P < 0.01 and ***P < 0.001; linear mixed model with factors reward (HiRe, LoRe) and monkey (M1, M2)]. For details see results and Table 2.

**Figure 4:**
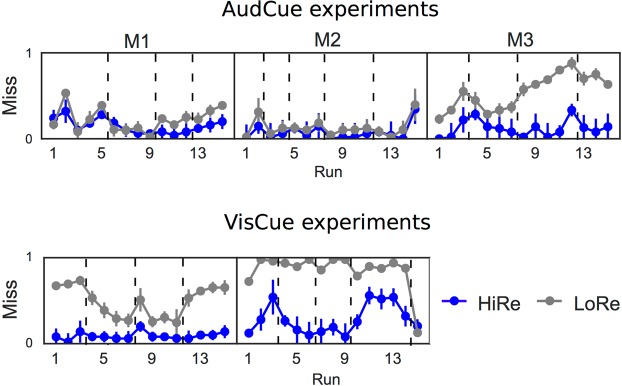
Temporal profile of the reward related performance effects. Dashed lines indicate the end of a daily session. Miss rate (MR) during the first 15 runs separately for each monkey with (top) auditory cues (M1, M2: AudCue1; M3: AudCue2) and (bottom) visual cues (see text for details). Error-bars indicate SEM.

**Figure 5:**
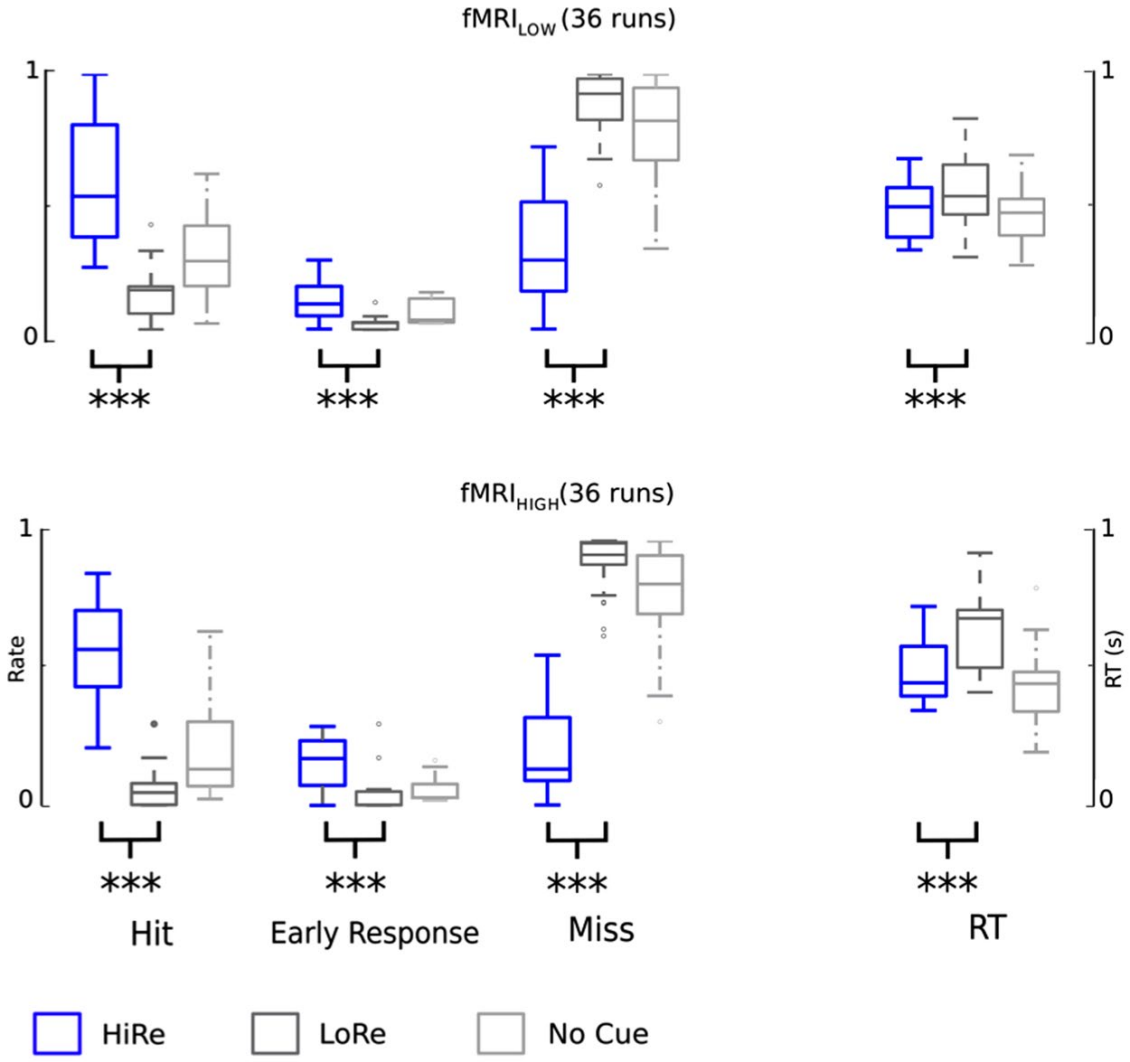
Performance during fMRI. Results are illustrated for fMRI_HIGH_ (Top) and in fMRI_Low_ (bottom). HiRe trials are depicted in blue, LoRe in gray and NoCue in light gray. Asterisks indicate significant differences between HiRe and LoRe trials (main effect of reward, *P < 0.05, **P < 0.01 and ***P < 0.001; linear mixed model with factors reward (HiRe, LoRe), monkey (M1, M2), and auditory wait signal (fMRI_HIGH_, fMRI_LOW_)]. For details see text and Table 3.

**Figure 6:**
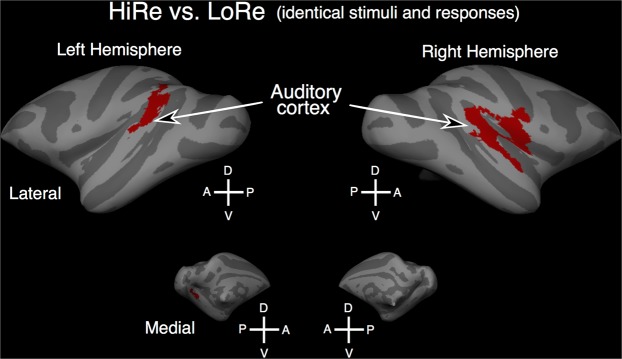
Brain areas showing stronger activation during HiRe than LoRe trials. Results are shown on inflated cortical surfaces (gyri: light gray; sulci: dark gray). The comparisons (Welch’s *v* test) were performed in surface space across 1^st^ level contrast parameter estimates and permutation inference was used to assess statistical significance (red clusters; HiRe vs. baseline and LoRe vs. baseline contrast parameter estimates, the runs of each monkey were treated as a permutation and variance group to accommodate heteroscedasticity, initial cluster-forming *Z* threshold 2.6, cluster-corrected P < 0.05). Note that the same P-value is attributed to the whole cluster in the PALM analysis, so differential P-value responses mapped to the brain should not be expected. Abbreviations: D dorsal, V ventral, A anterior, P posterior.

**Table 2:** Performance in AudCue1 and AudCue2.

Significant effects		Df	F	P
**Reward cue (HiRe**, **LoRe)** × **Experiment (AudCue1**, **AudCue2)** × **Monkey (M1**, **M2**, **M3)**				
HR	Reward cue	1,64	125	0.00002
	Reward cue × Monkey	2,64	62	1.0 × 10^–15^
	Reward cue × Monkey × Experiment	2,78	7.2	0.001
ER	Reward cue × Monkey	2,114	6	0.003
	Reward cue × Monkey × Experiment	2,110	12	0.00002
MR	Reward cue	1,61	162	8.0 × 10^–19^
	Reward cue × Monkey	2,62	54	2.6 × 10^−14^
	Reward cue × Monkey × Experiment	2,73	8.3	0.0006
RT	Reward cue	1,75	42	8.5 × 10^−9^
	Reward cue × Monkey	2,75	21	5.7 × 10^−8^
**M1**, **AudCue1: Reward cue (HiRe**, **LoRe)**				
HR	Reward cue (Hire > LoRe)	1,28	14	0.0008
ER	Reward cue (Hire > LoRe)	1,28	9.2	0.005
MR	Reward cue (Hire < LoRe)	1,28	33	3.6 × 10^−6^
**M2**, **AudCue1: Reward cue (HiRe**, **LoRe)**				
ER	Reward cue (Hire > LoRe)	1,24	24	0.00005
MR	Reward cue (Hire < LoRe)	1,24	36	3.5 × 10^−6^
RT	Reward cue (Hire < LoRe)	1,24	8.0	0.009
**M3**, **AudCue1: Reward cue (HiRe**, **LoRe)**				
HR	Reward cue (Hire > LoRe)	1,14	80	3.6 × 10^−7^
MR	Reward cue (Hire < LoRe)	1,14	26	0.00003
RT	Reward cue (Hire < LoRe)	1,14	22	0.0003
**M2**, **AudCue2: Reward cue (HiRe**, **LoRe)**				
HR	Reward cue (Hire > LoRe)	1,13	5.0	0.044
MR	Reward cue (Hire < LoRe)	1,13	6.1	0.028
RT	Reward cue (Hire < LoRe)	1,13	6.2	0.027
**M3**, **AudCue2: Reward cue (HiRe**, **LoRe)**				
HR	Reward cue (Hire > LoRe)	1,14	25	0.0002
MR	Reward cue (Hire < LoRe)	1,14	26	0.0002
ER	Reward cue (Hire < LoRe)	1,14	6.1	0.027
RT	Reward cue (Hire < LoRe)	1,14	20	0.0005

**Table 3:** Performance during fMRI.

Significant effects		Df	F	P
**Reward cue (HiRe**, **LoRe)** × **Auditory wait signal (Low**, **High)** × **Monkey (M1**, **M2)**				
HR	Reward cue	1,48	240	2.6 × 10^−20^
	Reward cue × Monkey	1,48	28	3.0 × 10^−7^
ER	Reward cue	1,47	47	1.4 × 10^−8^
	Reward cue × Monkey	1,48	21	0.00003
MR	Reward cue	1,75	786	1.7 × 10^−41^
	Reward cue × Monkey	1,75	21	0.00002
RT	Reward cue	1,36	23	0.00003
**M1: Reward cue (HiRe**, **LoRe)**				
HR	Reward cue (Hire > LoRe)	1,26	173	5.4 × 10^−13^
ER	Reward cue (Hire > LoRe)	1,23	957	3.0 × 10^−20^
MR	Reward cue (Hire < LoRe)	1,23	137	3.6 × 10^−11^
M2: Reward cue (HiRe, LoRe)				
HR	Reward cue (Hire > LoRe)	1,34	1036	4.7 × 10^−27^
MR	Reward cue (Hire < LoRe)	1,23	870	8.8 × 10^−20^

**Table 4:** Activation differences between HiRe and LoRe trials in STG ROIs.

Significant effects		Df	F	P
**Hemisphere**, **ROI**, **Parameter (Hit**, **Early response**, **Miss)**, **Reward cue (HiRe**, **LoRe)**, **Monkey (M1**, **M2)**				
	Reward cue	1,471	8.1	0.005
	Reward cue × Parameter	2,527	8.9	0.0002
	ROI × Reward cue × Parameter	6,573	2.9	0.009
	ROI × Reward cue × Parameter × Hemisphere	6,593	2.6	0.017
	Reward cue × Monkey × Parameter	2,110	12	0.00002
**Hemisphere**, **ROI**, **Reward cue (HiRe**, **LoRe)**, **Monkey (M1**, **M2)**				
Hit	Reward cue	1,80	6.8	0.011
	ROI × Reward cue × Hemisphere	3,170	9.2	0.005
Early r.	Reward cue	1,63	4.7	0.034
	ROI × Reward cue × Hemisphere	2,165	3.5	0.032
Miss	Reward cue × Hemisphere	1,135	7.0	0.009
	Reward cue × Monkey	1,68	8.0	0.020
**M2: Reward cue (HiRe**, **LoRe)**				
Miss	Reward cue	1,35	4.2	0.048

**Figure 7:**
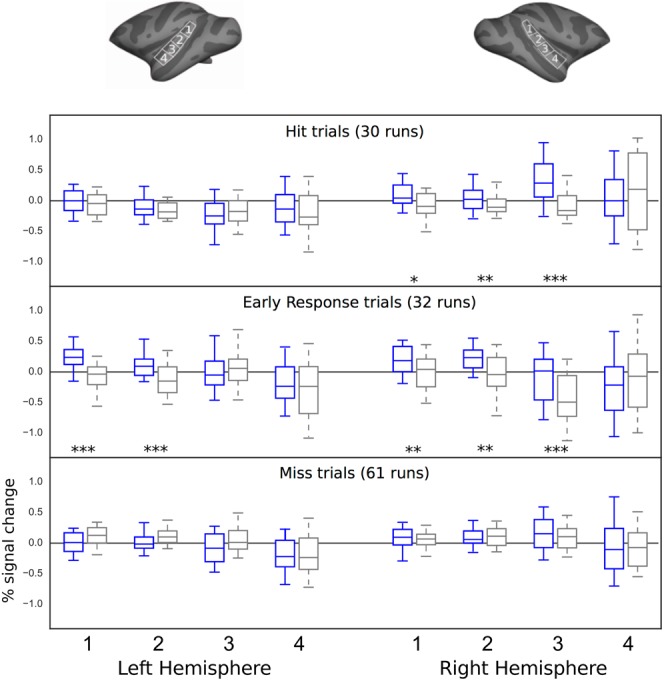
Region-of-interest (ROI) analysis of activation differences between HiRe and LoRe trials in monkey STG. The box plots show mean signal magnitudes (two monkeys) in each anatomically-defined STG ROI. To remove outliers, the central 85% of values were included in each case leaving 15–30 runs for M1 and 15–30 runs for M2, depending on the response measure in question. Asterisks indicate significant pair-wise tests comparing signal magnitude between HiRe and LoRe trials in each ROI (permutation-based significance testing using Welch’s *v* tests, two-sided, 10 000 permutations, FWER corrected across all pair-wise comparisons, *P < 0.05, **P < 0.01 and ***P < 0.001). Note that each test compared activation across HiRe and LoRe trials with identical auditory stimuli and motor responses. The inserts at top show the location of the ROIs. The difference between HiRe and LoRe trials was significant only during the hit and early response trials and the effects were most consistent in the posterior and middle parts of STG.

### Psychophysiological interactions analysis

We conducted a psychophysiological interactions (PPI) analysis to investigate which brain regions were functionally interacting with auditory cortex during the present task. First-level analysis was conducted using a model with psychological regressor (contrast HiRe > LoRe, hit and early response trials), physiological regressor (mean timeseries in bilateral primary auditory cortex, ROIs; based on tonotopic measurments during fMRI in at least 3 monkeys), and PPI (interaction between psychological and physiological regressors) as explanatory variables. The model also included all the rest of the explanatory variables of the original model (see *fMRI data analysis*). Higher level analysis was conducted in voxel space using FSL’s PALM^30^; version alpha26; 10 000 permutations). Finally, the results were resampled to the cortical surface of the template monkey brain for visualization^27,29^.

## Results

### Behavioral results in AudCue1, AudCue2 and VisCue experiments

The present Incentivized Attention Paradigm (IAP paradigm) is illustrated in Fig. 1. We first looked at the impact of the reward cues on performance in this paradigm. Mean performance across each run and animal in the AudCue1 and Audcue2 experiments is shown in Fig. 2. Note that as the task is not a discrimination nor a categorization task, d’ cannot be computed here. Hit and miss rates in the current task are, however, comparable or better than those reported in previous monkey studies^12,13,31^. To test whether the reward manipulation (HiRe vs. LoRe) significantly modulated performance in the AudCue1 and AudCue2 experiments, we used linear mixed models (intercept for run) with repeated measures factor reward cue (HiRe, LoRe) and fixed factors of experiment (AudCue1, AudCue2) and monkey (M1, M2, M3). Each performance parameter (hit rate HR, miss rate MR, early response rate ER, and reaction time RT) was analyzed using separate models. Significant reward cue main effects were observed (Table 2), seen as higher HR, lower MR and faster RT in the HiRe than LoRe trials. The linear models also revealed significant reward cue × monkey and reward × experiment × monkey interactions indicating that the effects associated with the reward cue manipulation were not identical in the two experiments and three monkeys. To understand the source of these interactions, we analyzed performance separately in each monkey and experiment (linear mixed model, intercept for run, repeated measures factor for reward cue; significant effects are listed in Table 2). These analyses showed that one of the monkeys (M3) had high performance for the HiRe trials. The other two monkeys showed less robust, but still significant preferences for the HiRe trials. Importantly, these analyses verified that each monkey showed significant reward cue effects in AudCue1, AudCue2 or both. However, significant differences between HiRe and LoRe trials were not always observed in the same performance measures across the monkeys and experiments, which explains the interactions with the monkey factor observed in the overall analysis.

Correspondingly, performance in the VisCue experiment (Fig. 3) was analyzed using linear mixed models with repeated measures factor reward cue (HiRe, LoRe) and fixed factor monkey (M1, M2). These analyses showed significant reward cue main effects for HR (F_1,46_ = 315, P = 3.3 × 10^−22^; HiRe > LoRe), MR (F_1,46_ = 144, P = 9.1 × 10^−16^; HiRe < LoRe) and RT (F_1,46_ = 33, P = 6.9 × 10^−7^; HiRe < LoRe). In contrast to AudCue1 and AudCue2 experiments, no significant reward cue × monkey interactions were observed, thereby there were no significant differences in performance between the two monkeys.

### Temporal profile of performance

Figure 4 shows the miss rate (MR) based results. This parameter was chosen because it showed the most consistent reward-cue effects across monkeys and behavioral experiments. In the AudCue experiments (top), the difference between HiRe and LoRe trials emerged within the first 15 testing runs consisting of 100 trials each. Note that the results are shown either for the AudCue1 or AudCue2 experiment depending on which one was performed first (M1 and M2 started with AudCue1, M3 started with AudCue2). In M1, lower MR in HiRe than LoRe trials is observed from the 10^th^ run onward (after ~1000 trials). M2 showed consistently better performance (i.e., lower MR) already during the first few runs (after a few hundred trials), but the difference between HiRe and LoRe in this monkey, although seen on some runs with the auditory cues, was not systematically observed in all runs. M3 learned the difference between HiRe and LoRe cues already during the first run (first hundred trials). Note that in M1 and M3 the reward cue effects on MR were observed consistently after 100 (M3) or 1000 (M1) trials. In the VisCue experiment (bottom), the two monkeys that were tested on this experiment (M1 and M2) both showed a distinct difference between the HiRe and LoRe trials already during the first run. This faster learning of the visual cues could relate to the fact that the VisCue experiment was conducted after the auditory cue experiments.

### fMRI experiment: Behavioral results

Because the VisCue experiment showed stronger effects than the AudCue experiments, the VisCue paradigm was chosen for fMRI. Similar to the VisCue experiment conducted outside the scanner, performance during fMRI (Fig. 5) was better in the HiRe than LoRe trials. However, in contrast to the behavioral experiments, there were more early responses during HiRe than LoRe trials during fMRI with the ER measure showing a significant effect. Interestingly, this effect is opposite to that found in previous studies using similar reward incentive paradigms in monkeys where the monkeys maximized their reward by increasing their ER rates for LoRe trials^15,18,25^. This was probably, at least partly, because during fMRI the trials were continued until the volume acquisition had completed (Fig. 1B). Thus, in the scanner, it was not possible for the monkeys to stop a LoRe trial with an early response. This constraint on the task timing during fMRI resulted in very low ER and high MR in LoRe trials. The relatively higher ER during the HiRe trials, in turn, might relate to the monkeys being impatient to receive the large juice reward. Note, that to maximize reward, the best strategy for the monkeys would have been to give more early responses for LoRe trials. However, instead we see that the monkeys gave more early responses for HiRe trials, thus they were not working to maximize reward in the same manner as seen in previous studies^15,18,25^.

As in the analysis of performance data in the behavioral experiments (above section), we tested the reward cue effects using linear mixed models with repeated measures factor reward cue (HiRe, LoRe), and fixed factors auditory wait signal (low, high) and monkey (M1, M2). The results are summarized in Table 3. The performance during the trials without cues (20% of the trials) is depicted as light gray in Fig. 5. Note that all behavioral parameters showed a significant main effect of reward cue. The analyses also revealed significant monkey × reward cue interactions for HR, ER, and MR suggesting that the monkeys performed the task using slightly different task strategies: M1 had a higher ER for HiRe trials than M2. Importantly, there were clear effects for both monkeys with HR and MR (Table 3).

### fMRI results

We hypothesized that due to the reward cue manipulation the monkeys would focus more strongly on the auditory wait signal during HiRe than LoRe trials and that this would result in higher fMRI activation in auditory cortex during HiRe than LoRe trials. To test for activation differences between HiRe and LoRe trials, we first analyzed activation in the 19 (out of 72) runs that contained all HiRe and LoRe trial types (i.e., hit, early response and miss trials). A run that e.g. did not contain any HiRe miss trials was excluded from this analysis. Supporting our initial hypotheses, contrasts between HiRe and LoRe trials in these runs showed enhanced activation in HiRe trials throughout broad STG regions bilaterally. Higher activation during HiRe than LoRe trials was also observed in right opercular cortex and in left extrastriate occipital regions (Fig. 6). No regions showed higher activation during LoRe than HiRe trials.

We were also interested in the effects of the reward cue manipulation on activations in tonotopically organized regions of AC. Therefore, we compared activation in the runs with the high-pitch (fMRI_high_) and low-pitch auditory wait signal (fMRI_low_) using separate contrasts and two-way ANOVAs with the factors pitch of auditory wait signal (fMRI_High_, fMRI_Low_) and reward cue (HiRe, LoRe). Because of generally weak tonotopic effects with the two pitches, these analyses yielded no significant topographic effects related to the tonotopic axis.

### fMRI ROI analyses

To understand the source of the reward cue effects on the activations in auditory cortex, we conducted ROI analyses using similar ROIs as in a previous comparative study on audio-visual selective attention^13^. To remove outliers, the central 85% of values were included in each case leaving 15–30 runs for M1 and 15–30 runs for M2, depending on the parameter in question. Figure 7 shows the comparison of mean signal magnitude in each ROI during HiRe and LoRe hit, early response and miss trials. This figure also summarizes the results of separate tests comparing signal magnitude between HiRe and LoRe trials in each ROI (permutation-based significance testing using Welch’s *v* tests, two-sided, 10 000 permutations, FWER corrected). Note that each test compared activation across HiRe and LoRe trials with identical auditory stimuli and motor responses. Significant reward cue differences were observed in hit and early response trials, but not in miss trials.

To investigate whether the reward cue effects show systematic differences across hemispheres, ROIs and the two monkeys, the ROI data were submitted to linear mixed models analyses. An omnibus analysis with factors hemisphere (left, right), ROI (1, 2, 3, 4), performance (hit, early response, miss), reward cue (HiRe, LoRe) and monkey (M1, M2), including an intercept for each run, revealed significant main effects for reward cue and the following interactions: reward cue × performance, ROI × reward cue × hemisphere, and ROI × reward cue × hemisphere × performance (Table 4).

Next, to better understand the bases for these interactions, we analyzed hit, early response and miss trials using separate models (i.e., different linear mixed models with the factors hemisphere, ROI, reward, and monkey; Table 4). In hit trials, there was a significant reward cue main effect across all ROIs and both hemispheres. There was also a three-way ROI × reward cue × hemisphere interaction with reward cue modulation during hit trials being stronger in the right posterior ROIs in comparison to the left (1–3, Fig. 7). Early response trials also showed an overall significant reward cue main effect. Additionally, a three-way ROI × reward cue × hemisphere interaction was observed because the reward cue effect in the middle ROI (3) was only significant in the right hemisphere (Fig. 7). In the miss trials, there was no main effect of reward. However, there were reward cue × hemisphere and reward cue × monkey interactions. The reward cue × hemisphere interaction was because miss-related activation tended to be higher during LoRe than HiRe trials in the left hemisphere, whereas the opposite trend was observed in the right hemisphere. The reward cue × monkey interaction during the misses, in turn, was because there were no significant differences between HiRe and LoRe trials for M1, but M2 showed higher activation during LoRe than HiRe miss trials (Table 4). The pairwise comparisons between HiRe and LoRe miss trials showed no significant effects in any of the ROIs.

In summary, the overall pattern of results is consistent with the hypothesis that reward cues modulate auditory attention within the simple auditory task, which generally caused broad modulation of monkey auditory cortex. Notably, unlike the systematic reward related effects for hit and early response trials (Table 4 and Fig. 7), no consistent HiRe vs. LoRe differences were observed during miss trials when the monkeys were likely paying less attention.

### Additional ROI analyses

As there were more HiRe than LoRe hit trials per run, we conducted further analyses for the eight auditory cortex ROIs to determine if this imbalance affected the fMRI ROI results. We tested whether the mean signal magnitude in each fMRI scanning run in either the HiRe or the LoRe trials correlated with the mean HiRe or LoRe HR of that run respectively. This analysis showed no significant correlations in any of the ROIs (HiRe: −0.22 < r < 0.018, 72 runs, P > 0.061 in all ROIs; LoRe: −0.127 < r < 0.260, 36 runs, P > 0.125 in all ROIs). Moreover, non-linearly transforming the HRs (second degree) to look at possible higher order effects also did not appear to impact on the fMRI ROI results, yielding nonsignificant results (HiRe: −0.18 < r < 0.06, 72 runs, P > 0.131 in all ROIs; LoRe: 0.015 < r < 0.158, 36 runs, P > 0.358 in all ROIs). Thus, the amount of hits achieved by the monkeys per run does not explain the difference between HiRe and LoRe hit trials shown in Fig. 7.

### Psycho-physiological interactions

**Figure 8:**
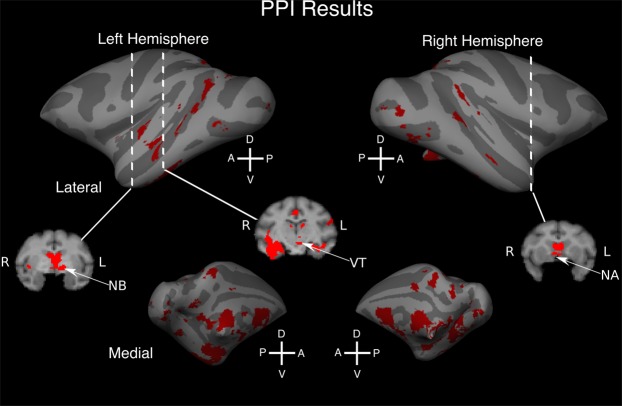
Brain areas showing significant psycho-physiological interactions (PPI) with the primary auditory cortex (A1, R and RT). These PPIs were analyzed by calculating the interaction between the difference between HiRe and LoRe hit and early response trials and the timeseries of the auditory cortical regions (see Methods). The analysis was performed across 1^st^ level contrast parameter estimates using permutation inference (10 000 permutations, the runs of each monkey were treated as a permutation and variance group to accommodate heteroscedasticity, initial cluster-forming *Z* threshold 2.6, cluster-corrected P < 0.05). The results (red clusters) are shown on inflated cortical surfaces (gyri: light gray; sulci: dark gray). We also show three coronal slices registered to a macaque standard brain in stereotactic coordinates^28^. Dashed lines on the lateral surfaces indicate the approximate position of the coronal slices in the brain. Abbreviations: D dorsal, V ventral, A anterior, P posterior, NB nucleus basalis, NA nucleus accumbens.

The results of the PPI analysis (Fig. 8) revealed that during the reward cue modulation, there was a significant functional interaction (initial cluster forming threshold 2.6, corrected P < 0.05) between primary auditory cortex and other cortical regions such as higher level auditory cortex, visual cortex, hippocampus, parietal cortex and medial frontal regions. Notably, significant effects were observed also in basal forebrain areas associated with dopaminergic (nucleus accumbens) and acetylcholinergic (nucleus basalis, thalamic nuclei and basal ganglia) modulatory influences (see Table 5 for a comprehensive list of regions).

**Table 5:** Regions showing significant (initial cluster forming threshold Z < 2.6, corrected p < 0.05) psycho-physiological interactions with the primary auditory cortex.

Name	Left	Right
**Cortical regions**		
Auditory koniocortex, lateral part		x
V1	x	x
V2	x	
V3	x	x
V4	x	x
V5		x
Parietal area PE	x	x
Parietal area P0		x
Area PGM	x	x
Area 23b	x	x
Superior temporal sulcus	x	x
Temporal area TE	x	
Temporal area TH		x
Orbital periallocortex		x
Hippocampus (including Ca1, Ca2, Ca3 and Ca4)	x	x
Entorhinal cortex		x
**Subcortical regions and the Cerebellum**		
Cerebellum	x	x
Periaqueductal gray	x	x
Inferior colliculus	x	x
Superior colliculus	x	x
Parasubiculum		x
Ventral tegmentum	x	
Medial geniculate nucleus		x
Paraventricular thalamic nucleus	x	x
Lateral reticular nucleus	x	x
Lateral dorsal thalamic nucleus	x	x
Mediodorsal thalamic nucleus	x	x
Ventral lateral thalamic nucleus	x	x
Anterior pulvinar	x	
Lateral pulvinar		x
Medial pulvinar	x	x
Centrolateral thalamic nucleus	x	x
Nucleus basalis	x	
Ventral pallidum	x	x
Nucleus accumbens		x
Globus pallidus	x	x
Caudate nucleus	x	x

These functional interactions in the identified brain areas were assessed alongside the reward related modulations in the auditory cortical regions showing main effects (Fig. 6) in relation to the macaque brain atlas in stereotactic coordinates^28^.

## Discussion

Remarkably, in the present paradigm (Incentivized Attention Paradigm; IAP), after acquiring the basics of behavioral training and acclimatization (e.g., chair training, responding to sounds with the lever and accepting reward, which takes about 2–3 months per monkey), the monkeys showed systematic behavioral effects within a few task sessions. By comparison, in previous monkey auditory studies using active listening tasks, training has typically required several months or a few years of daily training sessions and many thousands of trials^13,32–39^.

Monkeys performed a very simple auditory attention-engaging task in which they were required to withhold a response during a wait signal and make a response after an auditory go signal in order to receive a juice reward. The auditory task required no motor-response selection, abstract task instruction, or other demanding training components that are difficult and time consuming on which to train nonhuman animals. Moreover, auditory task performance was modulated using the high and low reward incentive cues, which the monkeys readily learned to discriminate from each other without specific training. Significantly different performance between HiRe and LoRe trials emerged within a few runs of 100 trials in all of the tested conditions. This difference was more pronounced and systematic during the VisCue (where HiRe and LoRe cues consisted of two distinct visual patterns) than AudCue (two distinct sounds) conditions.

After visual reward cue onset in the VisCue condition, an auditory wait signal was presented indicating the monkeys should withhold the response until an auditory go signal occurred. This was designed to redirect attention from the visual to the auditory modality to help the monkeys to detect the auditory go signal. As expected, we found that activation in auditory cortex timed to the auditory wait signal was higher during HiRe than LoRe trials. This effect is not due to auditory stimulation as identical sounds were presented during both types of reward trials.

The results also alleviate concerns about potential confounds, such as visual stimulation or motor responses contributing to the HiRe-LoRe difference observed in auditory cortex. For instance, although a different visual cue was used in the HiRe versus LoRe trials, the activation difference in auditory cortex is not due to visual stimulation. This is evident in the results shown in Fig. 7 in which a significant HiRe vs. LoRe difference is observed only during hit and early response trials. The enhanced activation during HiRe hit or early response trials is also not due to general task-performance (e.g., motor response) differences because all HiRe vs. LoRe comparisons were conducted across trials with similar performance (e.g., hit HiRe vs. hit LoRe trials; see Fig. 7). Furthermore, the effect of reaction time differences (in hit and early response trials) was controlled for in the (first-level) analysis, and we confirmed that non-linear RT effects and the higher number of hits in HiRe than LoRe trials did not significantly modulate signal magnitudes in the ROIs. Thereby, the overall findings support the initial hypothesis that the reward incentive cues affected monkeys’ motivation to perform the auditory task and that this modulated activation in monkey auditory cortex.

As a point of reference, in our previous fMRI study on audio-visual selective attention, monkeys were trained to perform a spatial discrimination task attending to stimuli in one sensory modality while ignoring those in the other^13^. In that study despite the extensive training over two years, we found that the monkeys’ frequent attention lapses significantly altered the pattern of attention-related activations, complicating cross-species comparisons to humans. In the present study, to decrease the amount of training, we relied on incentive cues and used a simple auditory task. Comparisons were conducted across trials with focused performance, thus we were able to control for the effects of lapses in attention. Interestingly, here we observed that the HiRe hit and early response (but not miss) trials were associated with enhanced activation in broader stretches of auditory cortex. The present findings are an important indication in nonhuman animals that an active auditory task, minimizing the effects of lapses in attention, can broadly modulate auditory cortical responses as it is known to do with humans^1–3,13,19–21^. However, to achieve this required using reward incentive cues, which is worth considering further, because it is a crucial facet of the paradigm.

Is it possible that the modulations in monkey auditory cortex were primarily due to reward expectancy and not due to auditory attention? The way the paradigm was designed and the behavioral and fMRI results support the notion that both are involved, as we now consider.

Recent studies have shown that monkeys have an extraordinary ability to make reward associations to disparate visual objects and to retain these associations for months^40,41^. These object reward associations have been found to modulate activations in both frontal regions and high-level visual regions^41^. Previous macaque studies have also shown reward expectancy modulations of neuronal responses and fMRI activation in both visual^42^ and auditory cortex^43–45^. In the present study, the HiRe trials were associated with stronger activity than the LoRe trials primarily in broad auditory cortical regions and, as we also show, this involves interaction between auditory cortex and sites along both dopaminergic and cholinergic pathways.

It is also known that the systems for motivation and attention are not functionally fully separable or at least can interact and jointly influence sensory cortices. Also, dopaminergic^46^ and acetylcholinergic^47–51^ neuromodulatory systems, implicated in reward processing and attention, affect auditory cortical neuronal processing. One could even argue that reward expectancy is an integral component of focused attention^52–55^ and, in fact, the results of most animal studies regarding auditory attention could be seen as reward expectation related modulation of sensory processing^52,56^. Thus, the specific effects of reward or attention are difficult to segregate, at least at the level of whole-brain imaging. Moreover, our PPI results indicate that it is likely that both systems affect sensory cortex, given that both regions associated with acetylcholinergic (nucleus basalis) and dopaminergic (accumbens) modulations interacted with the effects in auditory cortex.

Thereby, inasmuch as only attention-related influences cannot explain the results, our results are also unlikely to be explained solely by reward expectancy effects, for the following reasons: First, in the previously mentioned studies^42–45^, reward value and thus reward expectancy effects were directly linked to specific visual or auditory stimuli. In contrast, in the present study after the reward cues, identical wait and target sounds were associated with either high or low reward and the fMRI volume was timed to these sounds. Second, any reward expectancy effects in the present study should have equally affected auditory cortex activations during the miss trials. However, no systematic activation differences between the HiRe and the LoRe trials were observed during miss trials, suggesting that the monkeys lost their focus and missed both the auditory wait and go signals. Third, during fMRI, the monkeys showed more early responses (resulting in trial termination and no reward) to HiRe rather than LoRe trials, wheareas animals working to maximize reward as seen in other studies^15^ would predict more early responses to LoRe trials. Finally, previous human studies using MID paradigms have shown that reward cue based paradigms can be effectively used to guide visual attention in humans, having a remarkably similar impact on sensory cortical processes as traditional attention tasks^57–62^. We now show similar effects on sensory cortex for the first time in nonhuman animals.

In the present study, the most distinct effects are seen in auditory cortex rather than in prefrontal regions implicated in value-based decision making^63^. Prefrontal areas, such as orbitofrontal  cortex did, however, show significant PPI results (Table 5), suggesting that these areas interact with auditory cortex in the current task. The lack of strong prefrontal cortex effects in Fig. 6 likely stems from the main whole-brain HiRe vs. LoRe comparisons summarizing effects across runs that contain all trial types (i.e., hit, early response and miss trials). Medial and orbito-frontal regions become prominent when value-based decisions require analysis of specific aspects of reward valuation or outcome^64^, which was not our main objective here. The use of this task design and fMRI volume acquisition paradigm, for the reasons noted in the previous paragraph, may also be why we found positive BOLD influences in sensory cortex. Two other studies using manipulations of reward report either decreases in auditory cortical neuronal responses as a function of reward expectancy^45^ or negative BOLD in visual cortex as a function of the amount of reward delivered on a previous trial^42^. However, results obtained using different neurobiological measures or in different sensory modalities cannot be directly compared or such comparisons should be made with caution pending future direct testing.

The advantage of the IAP paradigm is that systematic auditory behavioral manipulations and significant brain modulation can be achieved extremely quickly. Moreover, the results show that the monkeys learned to reverse the reward relationship in AudCue1 and AudCue2 within a few trials. Thus, with this task the monkeys quite flexibly reinterpret the behavioral meaning of the cues and stimuli. By comparison, training in traditional paradigms often requires 10 s of 1000 s of trials and typically by that point the animals have stereotyped their behavioral response patterns (making it difficult to reinterpret cue and stimulus conditions) and the results suffer from regular lapses of attention^12,13^. IAP requires the use of reward incentive cues and as such it is not a traditional attention paradigm. However, the impact on sensory cortex by incentivized attention is actively being studied. This study contributes to these efforts by identifying the functional imaging impact on sensory cortex by reward-driven attention and the neuromodulatory sites involved.

The IAP could in the future be further developed and combined with other approaches to more selectively manipulate the focus of attention. Namely, the use of incentive cues in the IAP paradigm serves to interest the monkeys in the trial if a high immediate reward can be expected. In turn, the auditory wait signal helps to direct the general focus of the monkey’s attention to sounds. Within the context of the visual cue paradigm, for example, using a mixture of auditory wait signal features associated with specific reward cues might be successful in manipulating the animals’ within-sensory-modality selective attention (e.g., attend to a particular feature of the auditory wait signal mixture to receive the high reward). The paradigm could also be hybridized with a more traditional paradigm, whereby animal task training is expedited and more quickly leads to testing the animals on cognitive tasks that gradually eliminate the reliance on the incentive cues (in weeks rather than months or years). These and other further developments to innovate training on cognitive tasks in nonhuman animals could provide an important foundation for being able to better translate the insights obtained in animal models to humans to advance scientific knowledge on the neural bases for cognition.

Due to the co-evolution of language and cognition in humans, some have argued that the extent to which nonhuman primates can model human auditory cognition remains unclear^10,11,32^. However, it is possible that challenges in training monkeys to maintain motivation and to perform stably on auditory tasks has influenced this impression^12,13^. Our results show that when the task is differentially incentivized and thus becomes more relevant for the monkeys, task training can be accomplished in a relatively short amount of time *and* that, similar to humans, active listening results in substantial activation modulations in broad regions of auditory cortex. The correspondence between our neuroimaging findings in macaques and related observations in humans altogether support the notion that substantial segments of the human auditory neurocognitive system are, at least qualitatively, based on evolutionarily conserved functionality.

## Data Availability

The data used to generate the figures are shared using the Open Science Framework under Laboratory of Comparative Neuropsychology (https://osf.io/arqp8).
